# Catastrophic chromosome fragmentation probes the nucleoid structure and dynamics in *Escherichia coli*

**DOI:** 10.1093/nar/gkac865

**Published:** 2022-10-16

**Authors:** Tulip Mahaseth, Andrei Kuzminov

**Affiliations:** Department of Microbiology, University of Illinois at Urbana-Champaign, Urbana, IL, USA; Department of Microbiology, University of Illinois at Urbana-Champaign, Urbana, IL, USA

## Abstract

*Escherichia coli* cells treated with a combination of cyanide (CN) and hydrogen peroxide (HP) succumb to catastrophic chromosome fragmentation (CCF), detectable in pulsed-field gels as >100 double-strand breaks per genome equivalent. Here we show that CN + HP-induced double-strand breaks are independent of replication and occur uniformly over the chromosome,—therefore we used CCF to probe the nucleoid structure by measuring DNA release from precipitated nucleoids. CCF releases surprisingly little chromosomal DNA from the nucleoid suggesting that: (i) the nucleoid is a single DNA-protein complex with only limited stretches of protein-free DNA and (ii) CN + HP-induced breaks happen within these unsecured DNA stretches, rather than at DNA attachments to the central scaffold. Mutants lacking individual nucleoid-associated proteins (NAPs) release more DNA during CCF, consistent with NAPs anchoring chromosome to the central scaffold (Dps also reduces the number of double-strand breaks directly). Finally, significantly more broken DNA is released once ATP production is restored, with about two-thirds of this ATP-dependent DNA release being due to transcription, suggesting that transcription complexes act as pulleys to move DNA loops. In addition to NAPs, recombinational repair of double-strand breaks also inhibits DNA release by CCF, contributing to a dynamic and complex nucleoid structure.

## Graphical Abstract

**Graphical Abstract:**
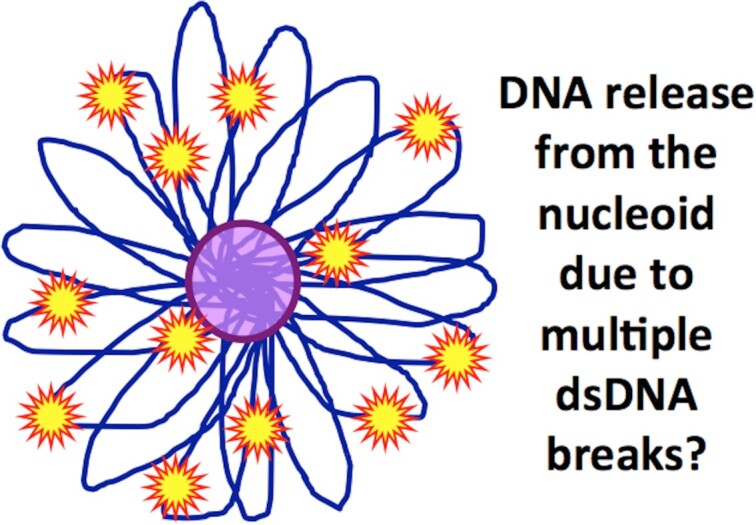
The currently accepted structure of bacterial nucleoid is tested by measuring release of free DNA after the chromosome suffers a hundred of double-strand DNA breaks.

## INTRODUCTION

The phenomenon of catastrophic chromosome fragmentation (CCF) was found serendipitously, when we were investigating the unexpected instability of hydroxyurea in aqueous solutions (1). In fresh solutions, hydroxyurea was characteristically bacteriostatic and weakly clastogenic; in contrast, aged solutions of hydroxyurea showed deep and acute killing, as if some toxic substances accumulated in hydroxyurea stocks upon storage. Moreover, the killing was associated with unusual levels of chromosome fragmentation (1). With typical DNA damage, chromosome fragmentation is only elevated in *recBCD* mutants, because these mutants do not repair double strand DNA breaks (DSBs) and do not degrade linear DNA (2). However, with aged hydroxyurea, fragmentation was high even in wild type (WT) cells. Gas chromatography–mass spectrometry analysis and *in vitro* experiments revealed that the toxic mix was a combination of hydrogen peroxide (HP), cyanide (CN) and nitric oxide (NO) (1). The synergistic toxicity of CN + HP and NO + HP combinations was already recognized in *Escherichia coli*, the killing proposed to be via enhanced DNA damage and double-strand breaks (3–6), but the overall chromosomal consequences of these treatments were unknown.

**Figure 1.**
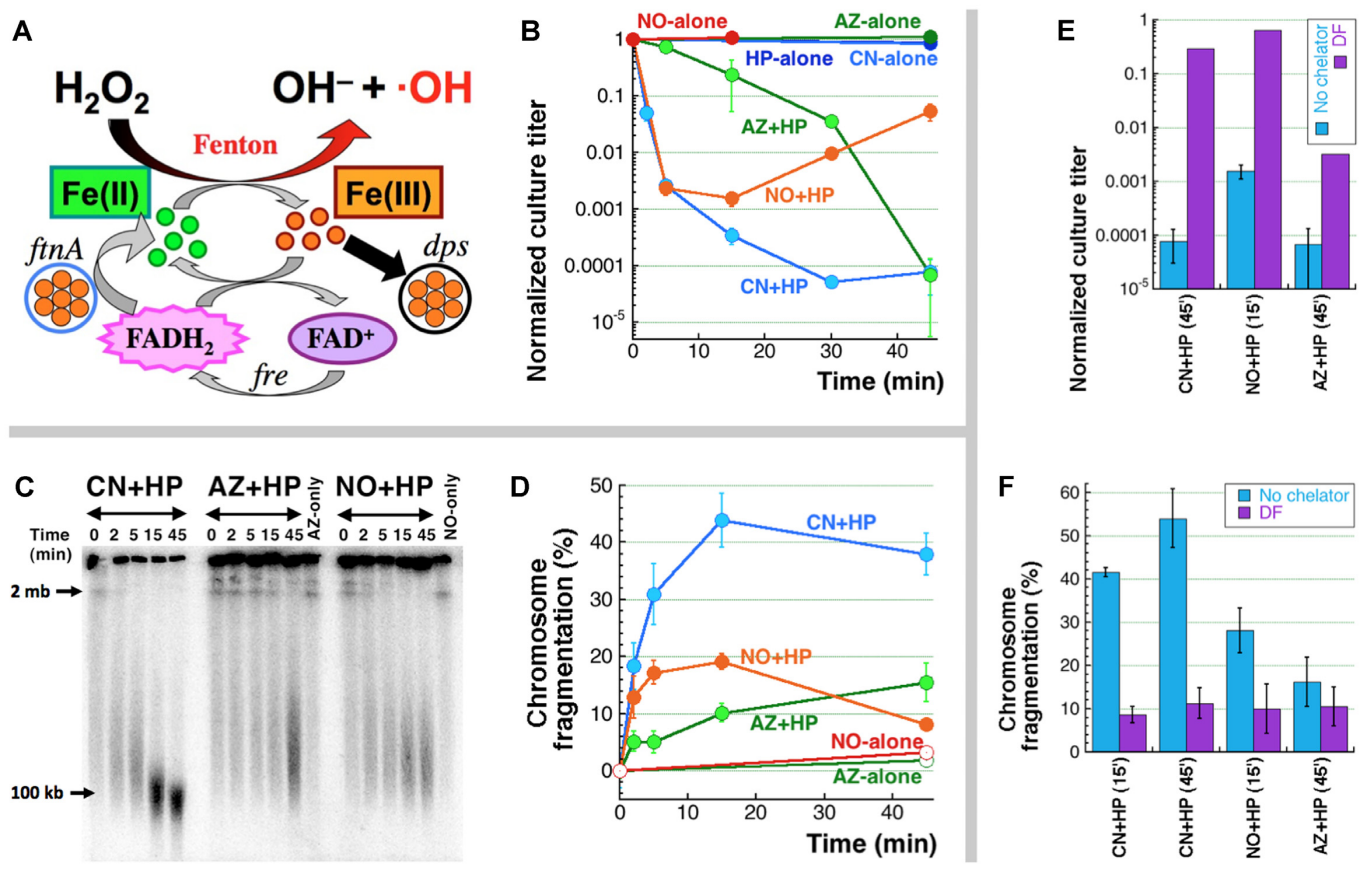
Potentiated HP toxicity via iron-dependent catastrophic chromosome fragmentation. (**A**) The iron side of Fenton reactants, prompted by CN and driven by FtnA and Fre, ensures a steady flow of Fe(II) to produce DNA damaging hydroxyl radicals from HP (H_2_O_2_). At the same time, at least a fraction of the resulting Fe(III) is sequestered by Dps. (**B**) Kinetics of survival of WT cells subjected to several synergistic or individual treatments. The synergistic treatments are: 3 mM CN + 2 mM HP (our standard treatment throughout the paper), 3 mM AZ + 2 mM HP, or 0.3 mM NO + 2 mM HP. Here and in the rest of the paper, all values are means of three or more independent measurements ± SEM, which means that if the bars are almost touching (even without overlapping),—the two means are not different. (**C**) A representative pulsed-field gel showing kinetics of chromosome fragmentation in WT cells subjected to CN + HP, AZ + HP or NO + HP treatments, as well as with AZ-only or NO-only treatments. (**D**) Quantitative kinetics of chromosome fragmentation from several gels like in ‘C’. (**E**) Survival of 45 min CN + HP treatment, 15 minute NO + HP treatment or 45 min AZ + HP treatment in the presence or absence of 20 mM deferoxamine (DF). (**F**) Quantification of chromosome fragmentation of WT cells subjected to either 15 or 45 min of CN + HP treatment, or to 15 min of NO + HP treatment, or to 45 min AZ + HP treatment, in the presence or absence of DF, from several gels like in ‘C’.

Synergistic killing with HP occurs via oxidative damage (7,8) and is medically-important, as it forms the basis of efficient killing of invading bacteria by neutrophils and macrophages (9–11). There are two modes of HP-killing in *E. coli* (6,12,13). Mode-one is observed in the 1–10 mM range of HP concentrations, it affects DNA repair mutants more than WT (in fact, acute 1–5 mM HP treatments do not kill WT *E. coli*) and it is completely blocked by *in vivo* iron chelators—indicating it to be due to the Fenton reaction (Fe(II) + H_2_O_2_ → Fe(III) + OH^—^ + ·OH) generating DNA-damaging hydroxyl radicals (Figure 1A). Mode-two HP-killing, observed at concentrations over 20 mM HP, is faster, kills independently of the DNA repair status of the treated cells and is insensitive to iron chelation (6,12,13). The agents like NO, CN, H_2_S and cysteine, synergize with HP and allow it to kill WT cells at 1–3 mM concentrations, which, as mentioned above, are bacteriostatic for HP-alone treatments (8).

Previously, we have characterized the chromosomal consequences of the CN + HP treatment, in which both HP-alone and CN-alone individual treatments are bacteriostatic, while the combined treatment kills fast (within an hour) and deep (by several orders of magnitude) (14). Imlay and Linn proposed that CN promotes the Fenton reaction by inhibiting respiration, and therefore, provides a source of reduced iron (Fe(II)), via increased NADH levels (15). Later it was reported that NADH accumulation allows flavin reductase, Fre, to boost the pools of reduced flavins, which would maintain the source of Fe(II) to support continuous Fenton chemistry (Figure 1A), thereby greatly increasing oxidative DNA damage (4,5). While confirming this general concept, we also tested a reasonable alternative, according to which CN inhibits catalases, making HP stable around cells,—but found no genetic evidence for (or against) this obvious scenario (14). Instead, our findings implicated ferritin, FtnA, as one of the sources of reduced iron in the presence of CN. At the same time, the Fenton reaction was actively limited by the miniferritin Dps, which sequesters iron in the presence of HP (Figure 1A) (14).

Importantly, in addition to these metabolic underpinnings of CN + HP toxicity, we found that what the CN + HP treatment induces in the chromosome is not merely elevated DNA damage, but such an unprecedented number of double-strand DNA breaks, that it literally shatters the entire chromosome into similar-sized (20–100 kb) pieces—a phenomenon that we call ‘catastrophic chromosome fragmentation’ (CCF) (14,16). The average size of the generated fragments (50 kb) translates into at least 100 DSBs per *E. coli* genome equivalent (∼5 Mb) and, if confirmed to reflect the real number of breaks *in vivo*, would explain the inability of even WT *E. coli* to survive this damage, as it should easily overwhelm the DNA repair capacity of a typical cell. Indeed, we found that, once CN is removed to restore ATP production, *E. coli* is still able to recover after ∼10 DSBs per genome equivalent, but is utterly incapable of recovering from and repairing the chromosome demise caused by ∼100 DSBs (16).

Since CCF is a novel phenomenon, several basic questions about it remain unanswered. For example, is CCF characteristic of only CN + HP treatment, or would other synergistic-with-HP treatments also induce it? The massive, unseen before scale of CCF raises suspicion of its artifactual nature, especially since our preliminary measurements showed no DNA loss due to subsequent degradation—the DNA loss expected if such a staggering number of double-strand DNA breaks were indeed generated in vivo. Theoretically, it is possible that, in combination with CN + HP chemistry, either DNA isolation procedure or DNA separation in pulsed-field gel electrophoresis (PFGE) introduce at least some of the breaks. We have observed such possibilities before, for example with RNase treatment during cell lysis (17), or during PFGE of linear DNA containing nicks (18,19),—warranting additional tests.

Another question was about the nature of the breaks themselves. The oxidative damage introduces nicks (single-strand breaks) into the backbone of the DNA,—either directly, or via base-excision repair of the oxidized bases (20,21). Thus, if the chromosomal fragmentation is due to oxidation, we must assume that it begins with single-strand DNA interruptions. Nicks in DNA are known to cause replication-dependent chromosome fragmentation by replication fork collapse (22,23). However, replication fork collapse, though efficient, is limited by replication itself, as the number of breaks cannot exceed the number of forks, and this number is modest even in rapidly-growing cells (24). Besides, replication-dependent breaks would result in chromosomal fragments of all sizes (23,25), and therefore, it cannot explain the observed uniform size of the fragments during CCF (14,16). Our previous experiments with DNA replication initiation-deficient conditions and mutants gave equivocal results, showing that half of the breaks were replication-dependent—implying that the other half was not (16).

Finally, if there are indeed so many breaks in the chromosomal DNA *in vivo*, they should release significant amounts of DNA from the nucleoid, the complex of bacterial chromosomal DNA with the nucleoid-associated proteins (NAPs) (26–28). Although appearing as a poorly-defined cloud in the middle of growing cells, the nucleoid in *E. coli* is actually structured as a dynamic helical ellipsoid (29), which in artificially-widened cells reveals its overall toroidal shape (30), organized by scaffold proteins like MukBEF condensin (31). When gently released from *E. coli* cells and spread, the nucleoid looks like a rosette of naked DNA loops anchored to the proteinaceous central scaffold (32,33). When sedimented in sucrose gradients, it shows a compact structure (34),—unless the DNA is nicked, as relaxed nucleoids sediment significantly slower (35). Interestingly, it takes about 50 nicks to make the nucleoid ‘completely relaxed’ by this sucrose gradient sedimentation assay, showing that individual loops are supercoiled independently of other loops and putting the minimal number of such loops (with likely additional internal structure) around 50 (35). While a similar approach using X-ray nicking produced a similar number (43 ± 10) of independently-supercoiled domains per *E. coli* chromosome (36), more recent measurements with reporter constructs put the number of independently-supercoiled domains around 400 (37,38),—perhaps detecting the internal structure of the main loops. Inhibition of DNA gyrase, the main type II bacterial topoisomerase, yields a maximum of 45 breaks per chromosome, interpreted as having reached the limit of one break per loop (39). Interestingly, a recent estimate of MukBEF condensin complexes (dimers of dimers), assumed to organize DNA loops, is also ∼53 per chromosome (31).

Electron microscopy pictures of the folded *E. coli* nucleoid generally supported the proposed structure (32,33,37,40,41). If the *E. coli* chromosome size is taken for 5 Mbp, 50 loops translate into 100 kb of DNA per loop. For example, poisoning of both gyrase and Topo IV, the other type II topoisomerase, releases slightly smaller, 50–100 kb DNA loops (42), generally confirming the ∼50-loop nucleoid structure. If such a structure is hit with 100 or more double-strand breaks, a significant amount of protein-free DNA is expected to be released from it, especially if breaks are targeted to the DNA-scaffold contacts.

In our previous work, we showed that the efficient killing by CN + HP is due to cyanide recruiting iron from the intracellular depots directly to chromosomal DNA (14), and further, due to its ability to promote the Fenton reaction of iron with hydrogen peroxide directly on the DNA (DNA self-targeting Fenton reaction), leading to generation of double-strand DNA breaks (16). We also showed that the bulk of CN + HP-induced primary DNA lesions are mended by base-excision repair and single-strand-break repair (16). Finally, we found that recombinational repair is inactive during the treatment, because cyanide blocks production of ATP (16), while all recombinational repair functions depend on ATP hydrolysis (2). As already mentioned above, once the treated cells are changed into a fresh medium, recombinational repair can mend ∼10 double-strand breaks per genome equivalent (16).

Thus, we have a clear picture of how the cell mends massive oxidative DNA damage and what are the reparable limits of it. At the same time, the mechanism behind CCF, as well as its nucleoid consequences, remained unexplored. Here we seek to answer four major questions: (i) Does CCF accompany other HP-based combined bactericidal treatments, or is it only observed after CN + HP treatment? (ii) Could the PFGE-detectable CCF be an artifact of chromosomal DNA isolation and/or separation methods? (iii) Are the DSBs generated directly or are they formed as a result of replication fork collapse at single-strand interruptions in the template DNA? (iv) How much DNA is released by CCF from the nucleoid structure, and what are the cellular processes, or the individual NAPs, that promote or hinder the DNA release?

## MATERIALS AND METHODS

### Strains and plasmids

*Escherichia coli* strains used are all K-12 BW25117 derivatives (43) except for *dnaA46*, *dnaC2* and *dut recBC* (Ts), which are in the AB1157 background. Alleles were moved between strains by P1 transduction (44). The mutants were all deletions from the Keio collection (43), purchased from the *E. coli* Genetic Stock Center, and were verified by PCR and phenotypically, whenever possible. For double mutant construction, the resident kanamycin-resistance cassette was first removed by transforming the strain with pCP20 plasmid (45).

### Reagents

Hydrogen peroxide and diethylamine NONOate were purchased from Sigma. Potassium cyanide and sodium azide were purchased from Fisher-Scientific.

### Growth conditions and viability assay

To generate killing kinetics, fresh overnight cultures were diluted 500-fold into LB medium (10 g of tryptone, 5 g of yeast extract, 5 g of NaCl, 250 μl of 4 M NaOH per liter (44)) and were shaken at 37°C for about 2.5 h or until they reached exponential phase (OD_600_ ∼ 0.3). At this point, the cultures were made 3 mM for CN and/or 2 mM for H_2_O_2_ (or the indicated treatment) and the shaking at 37°C was continued. In order to measure survival/revival in cells treated with CN and H_2_O_2_ for 45 min, the cells were spun down, resuspended in fresh LB and allowed to grow at 37°C post-treatment. Viability of cultures was measured at the indicated time points by making serial dilutions in 1% NaCl and spotting them by 10 μl on LB plates (LB medium supplemented with 15 g of agar per liter). The plates were developed overnight at 28°C, the next morning colonies in each spot were counted under the stereomicroscope, while still small. All titers have been normalized to the titer at time = 0 (before the treatment).

### Measuring chromosomal fragmentation by pulsed-field gel electrophoresis

This follows exactly our previous protocols (14,23,25).

### Southern hybridization of chromosomal fragmentation

At the indicated time-points, samples were collected in duplicates, and non-radioactive plugs were made and run on pulsed field gels, as described above. The plugs were taken out of the wells after the run, transferred to glass tubes, and both the pulsed-field gels and the plugs were washed with 0.25 M HCl, followed by 0.5 M NaOH, and finally, with 1 M Tris–HCl pH 8.0; each wash was 40 min long. The treated plugs were then placed on Amersham Hybond N+ (GE Healthcare) nylon membrane, covered with Saran wrap, and DNA was transferred by vacuum for 1–2 h, while the DNA from the treated pulsed-field gels were transferred overnight via capillary transfer. Next, the DNA was UV-crosslinked to the membranes and probed with ^32^P-labelled *ori*- or *ter*-specific probe. Hybridization was carried out overnight at 63°C in a 0.5M Sodium Phosphate (pH 7.4) and 5% SDS hybridization buffer. In the morning, the membranes were washed thrice with 1% hybridization buffer and rinsed with water just before covering them with Saran wrap and exposing to a PhosphorImager screen. The resulting signals were measured with a PhosphorImager (Fuji Film FLA-3000).

### Isolation of rRNA

We followed the RNAsnap procedure (46) to isolate rRNA from ^32^P labelled exponential cultures (OD_600_ approximately 0.3) grown at 37°C. The rRNA samples were precipitated out via ethanol reprecipitation (0.1 × 5 M NaCl and 2× ethanol were added to 1× volume of rRNA sample and mixed vigorously by vortexing. The samples were then spun down at 16 000g for 5 min, and the supernatant was completely removed via aspiration and the pellet was resuspended in TE) prior to running them on a 1.1% agarose gel at 60 V for 2 h. After the run, the gel was dried and exposed to a PhosphorImager screen. The resulting signals were measured with a PhosphorImager (Fuji Film FLA-3000).

### Measuring linear DNA degradation

This generally follows our previous protocol (47). Cells were grown overnight in 1 ml of LB and 10 μCi (methyl-^3^H)-thymidine at 28°C. In the morning, cells were spun down and washed thrice with 1 ml of LB, then diluted 200 times into 40 ml LB and were shaken at 37°C for 2 h. Cells were then treated with 3 mM for CN and 2 mM for H_2_O_2_ for an hour, still shaking at 37°C, after which the cultures were split in halves. CN and H_2_O_2_ were removed from one half via centrifugation, and the cells were resuspended in the same volume of LB (20 ml). Both halves were then shaken at 37°C for 2 h. At the indicated times, 4 ml aliquots were taken and mixed with 4 ml of chilled 10% trichloroacetic acid. Cells were collected by filtration and prepared for scintillation counting, as previously published (47,48).

### Measuring nucleoid disassembly

Overnight cultures were diluted 500-fold into 25 ml LB medium containing 25–75 μCi ^32^P and were shaken at 37°C for about two and a half h or until they reached exponential phase (OD_600_ ∼ 0.3). At this point, total DNA plugs were made of 300 μl aliquots of untreated cultures, as described above. The cultures were made 3 mM for CN and 2 mM for H_2_O_2_, and shaking at 37°C continued for 45 min. Following this, CN and H_2_O_2_ were removed by centrifugation, the cells were resuspended in the same volume of LB, and shaking at 37°C continued. To isolate nucleoid-free DNA, we used a modified version of the total plasmid isolation protocol (49). At the indicated time-points, 3 ml aliquots were spun down and resuspended in 50 μl of 30% Sucrose in 50 mM Tris–HCl (pH 8), 10 mM EDTA. 350 μl of 2% SDS was then added, the content of the tube was mixed by inversion and the tube was placed at 70°C for 5 min to accomplish a complete cell lysis. Once the suspension cleared, 100 μl of 5M NaCl was added and, while the tube was still warm, mixed thoroughly via inversions. The tube was chilled on ice for 1 hour to cause SDS-salt precipitation. The precipitate, also containing big cellular structures like nucleoids and most of the chromosomal DNA, was sedimented by centrifugation at 16 000g for 20 min (room temperature), after which the supernatant was transferred to a fresh tube. Nucleic acids remaining in the supernatant were precipitated with 1 ml of ethanol and inversion, and the samples were spun down at 16 000g for 5 min, after which the supernatant was aspirated, while the pellet was resuspended in 20 μl TE. To remove RNA and polysaccahrides, 30 μl of 6M LiCl was added, the tube was mixed by thorough vortexing and chilled on ice for 15 min. After another 16 000g × 5 min centrifugation, the supernatant was transferred to a fresh tube. Nucleic acids were precipitated with 100 μl ethanol, mixing and centrifugation as above, and the DNA was resuspended in 20 μl TE. 10 μl of each sample was used to run on a 1.1% agarose gel at 60 V for 3 h. The gel was then dried and exposed to a PhosphorImager screen. The resulting signals were quantified with a PhosphorImager (Fuji Film FLA-3000).

## RESULTS

### Chromosome fragmentation is induced by other treatments that potentiate HP toxicity

Is CCF observed whenever HP treatment is potentiated with another agent? Two more agents, sodium azide (AZ) and nitric oxide (NO), inhibit respiration like CN does (50–52), and therefore, are bacteriostatic individually (Figure 1B). Moreover, NO is known to potentiate HP toxicity (3,5) and, together with HP, to play a critical role in microbicidal power of our immune cells (9,10). Azide could also potentiate HP toxicity (it was reported to potentiate ‘complete Fenton’ (53)),—for example, because, like CN and NO (54), AZ is also a known inhibitor of catalases (55,56).

We tested the ability of NO + HP and AZ + HP combinations to kill *E. coli* via double-strand DNA breaks, comparing the results to the CN + HP effects (14,16). We found that the early kinetics of death was similar for NO + HP and CN + HP (Figure 1B). However, because NO is actively degraded in the cells (57), the killing by NO + HP stops rapidly, and the surviving cells partially recover at later time points,—suggesting exacerbation of killing upon plating of the liquid culture when NO is still present. AZ + HP, on the other hand, behaves very differently from CN + HP, showing a shoulder of resistance for about 25 min before succumbing sharply later on.

The chromosomal fragmentation observed for these synergistic treatments generally corresponds to their killing potential. In the case of NO + HP, we find that the fragmentation increases steadily at the early time-points, like that of CN + HP, yet the final fragmentation observed in NO + HP is significantly lower (Figure 1CD), matching the better survival of this treatment (Figure 1B). Similarly, AZ + HP shows maximum fragmentation at the last time-point, but again, it is much lower than after CN + HP treatment. This suggests that, in addition to NO instability, the mechanism of potentiation of hydrogen peroxide toxicity by these compounds may be significantly different from that by cyanide. We also tested whether cell death and chromosomal fragmentation were dependent on iron in NO + HP or AZ + HP, as is the case with CN + HP (4,14), by carrying out the treatment in the presence of an intracellular iron chelator, deferoxamine (DF), that does not inhibit the cell cycle (58). We confirmed that NO + HP co-toxicity did depend on iron (or the Fenton chemistry), however, loss in viability due to the AZ + HP treatment was only partially blocked by DF (Figure 1E, F). This indicates that the AZ + HP synergistic toxicity occurs at least in part via a distinct iron-independent mechanism, perhaps similar to mode-two HP killing, mechanisms of which remain unclear (59).

### Chromosomal DNA and rRNA degradation after CN + HP treatment

Since the chromosome fragmentation induced by NO + HP or AZ + HP was modest compared to CCF after CN + HP (Figure 1CD), there was a possibility that all three treatments yielded a similar lower number of breaks within cells, but the CN + HP chemistry was different enough from the other two to yield additional, artifactual breaks during DNA preparation and/or running of the gel.

**Figure 2.**
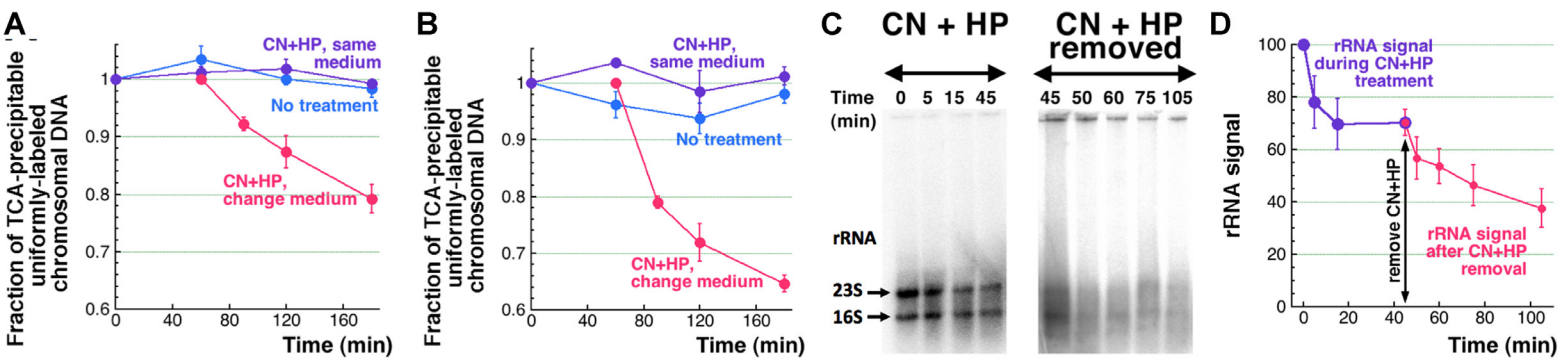
Removal of CN + HP treatment reveals subsequent ATP-dependent chromosomal DNA and rRNA degradation. (**A**) Kinetics of linear DNA degradation observed after WT cells were first treated for 60 min with CN + HP, after which they were changed into a fresh medium and incubated for two more h. An untreated control is also shown, as well as a culture treated continuously for 3 h. (**B**) Kinetics of linear DNA degradation observed upon removal of CN + HP after 60 min treatment from WT cells expressing extra RecBCD from the plasmid pDWS2. All treatments are like in ‘A’. (**C**) Representative agarose gels demonstrating the loss of rRNA from WT cells during the 45 min CN + HP treatment (left) and after changing the culture into a fresh medium and additional incubation for 60 min (right). (**D**) Kinetics of rRNA loss during the CN + HP treatment and upon subsequent changing into a fresh medium, from quantification of several gels like in ‘C’. CN + HP treatment gels (left) were all ran and quantified before we decided to extend the measurements after removing CN + HP (separate gels, right). To connect the two sets of results, all the ‘CN + HP-removed’ gels, besides the common time point of 45 min with CN + HP gels, also had 0 min of CN + HP treatment (for normalization),—omitted in ‘C’ for clarity. This duplicated the number of data for the 45 min CN + HP treatment/removed time point,—explaining its smaller error bar.

Perhaps the most direct confirmation that the chromosome fragmentation happens inside the cell during the treatment, rather than in the course of subsequent DNA isolation and manipulation *in vitro*, would be massive chromosomal DNA degradation still within the intact cells, as observed, for example, in gamma-irradiated *E. coli* cells (60). However, no chromosomal DNA degradation is observed even after 3 h during the CN + HP treatment (Figure 2A, B), as if no double-strand breaks were formed inside the treated cells. Yet, there is an apparent reason for this seemingly surprising result. Since linear duplex DNA degradation in *E. coli* is the function of the RecBCD helicase-nuclease, linear DNA stability in this case could be attributed to the fact that cyanide inhibits respiration and ATP-production in aerobic cells (61), as a result of which the ATP-driven RecBCD enzyme (62) can function only slowly.

Indeed, once we terminated CN + HP treatment by resuspending cells in a fresh medium, we detected significant linear DNA degradation (20% of the chromosome was degraded in two h) (Figure 2A), similar in the rate and extent to the chromosomal DNA disappearance observed after gamma-radiation (60). As reported before, at this level of CCF, removal of the treatment does not reveal any repair of double-strand breaks and does not influence the (extremely low) survival (16). Supplying additional RecBCD enzyme from a medium-copy-number plasmid pDWS2 (63) further increases the rate and extent of this degradation (35% of the chromosomal DNA is lost in two h) (Figure 2B). The modest increase in linear DNA disappearance in response to additional RecBCD suggests that the linear DNA degradation in WT cells is not significantly limited by the enzyme availability. Thus, double-strand breaks do indeed form inside the cells as a result of CN + HP treatment, but the fragmented chromosomal DNA is not degraded during the treatment because the CN-limited ATP production fails to support RecBCD function.

We have also noticed that ribosomal RNA (rRNA) appears to be relatively stable during CN + HP treatment, with ∼70% surviving the treatment (Figure 2C). The complex and/or compact structure of rRNA could be the reason behind this relative stability, which, in contrast, makes the duplex DNA uniquely sensitive to CN + HP-induced breakage. However, it was unclear whether rRNA did not suffer much (single-strand) breakage, or the breakage was extensive, but was absorbed by the stable structure of rRNA. We tested whether this apparent rRNA stability is due to the lack of ATP in the presence of CN by following rRNA stability in CN + HP treated cells after resuspending them in a fresh medium. We found that rRNA signal smears and weakens to less than 40% after the treated cultures are changed into the fresh medium (Figure 2C, D), not only demonstrating the considerable damage to ribosomes by the CN + HP treatment, but also showing that subsequent damaged ribosome recycling is an ATP-dependent process, likely because the complex ribosome structure has to be dismantled by RNA helicases.

### CN + HP treatment induces replication-independent two-ended breaks

Previously, we showed that blocking chromosomal replication using replication initiation-deficient mutants at non-permissive temperatures reduces CN + HP-induced CCF roughly by half, suggesting that at least one half of CCF is due to replication-dependent fragmentation (16), for example, due to replication fork collapse (RFC) at HP-induced DNA nicks (22). Even though CN inhibits respiration, it does not completely block the ATP production (61), and so slow replication continues even in the presence of CN—for example, see (64), where it is detectable even on ice. However, there was a possibility, which we never tested, that the non-replicating chromosome has a different structure,—for example due to the redistribution of NAPs in growing versus stationary cells (65),—which makes it less susceptible to the CN + HP-induced double-strand breaks. If true, then *all* CN + HP-induced breaks could happen away from replication forks, and so we needed to test replication-dependence of CN + HP-induced double-strand breaks directly.

**Figure 3.**
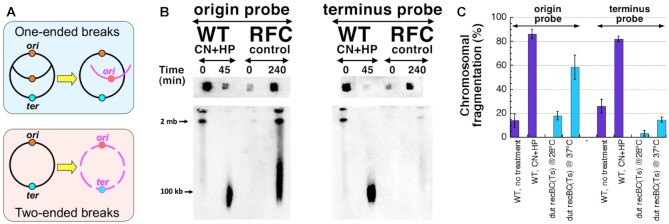
CN + HP-induced breaks are two-ended. (**A**) Hybridization with origin- versus terminus-specific probes can be used to distinguish between replication-dependent one-ended breaks due to replication fork collapse (RFC) (the top, blue frame) and direct, replication-independent, two-ended breaks (the bottom, pink frame). (**B**) A representative pulsed-field gel of *unlabeled* fragmented chromosomes, followed by gel-to-membrane transfer of DNA and blot-hybridization with origin-specific or terminus-specific probes, of WT cells treated with CN + HP. To facilitate transfer, the plugs were taken out of the wells after gel treatments and laid flat opposite their corresponding lanes. The *dut recBC*(Ts) strain was used as the ‘RFC control’ for replication-dependent one-ended breaks. (**C**) Quantification of hybridization of the CN + HP-fragmented chromosomes to the origin-specific or terminus-specific probes (compared to the *dut recBC* control for replication fork collapse) from several gels like in ‘B’.

Replication-dependent chromosome fragmentation yields only one-ended breaks, generated by demise of replication bubbles (Figure 3A, top). Therefore, the resulting sub-chromosomal fragments all carry the replication *origin* (Figure 3A, top). In contrast, the replication *terminus* would be either preserved on the circular part of the chromosome or occasionally end up on the longer-than-genome-equivalent linear chromosomes (under our PFGE conditions, these species pile up in the compression zone, marked ‘2 mb’ (Figure 1C)). Importantly, the terminus signal would be conspicuously absent among the subchromosomal fragments forming the fragmentation smear (23,66). This point is illustrated by Southern hybridization analysis of the chromosome fragmentation smear in the *dut recBC* mutant (the RFC control (Figure 3B)), where the entire smear hybridizes to the origin-specific probe, but does so poorly to the terminus-specific probe. This chromosomal fragmentation in the *dut recBC* mutant is all replication-dependent (23,66) and serves as a positive control for our analysis.

In contrast to the pattern of RFC, the uniformly-sized chromosomal fragments after 45 min of CN + HP treatment hybridize equally well to both the origin-specific and the terminus-specific probes in the chromosome fragmentation smear (Figure 3BC). It is important to note that 20% of the overall DNA signal still remains in the well,—and the terminus material could have been mostly there (see the RFC control as an illustration). Equal hybridization of both origin- and terminus-specific probes to the CCF smear demonstrates that the CN + HP-induced breaks are two-ended, therefore replication-independent (direct),—and also distributed over the chromosome rather uniformly (Figure 3A, bottom). The uniform distribution of the double-strand breaks over the entire chromosome means that some of them happen in the unreplicated part of the chromosome, making them theoretically irreparable and explaining the deep killing by CN + HP treatment. The reduction of CCF by 50% in the non-replicating chromosomes (16) then is likely due to a special condensation by one of the NAPs,—however, this NAP is yet to be identified.

### CCF does not release DNA from the nucleoid

Next, we asked whether these uniformly distributed double-strand DNA breaks were random, or they were associated with the chromosomal DNA attachments at the nucleoid scaffold. The chromosomal DNA in both bacteria and eukaryotes is organized as rosettes of radial loops (32,67). Such loops in *E. coli* start at the proteinaceous central scaffold in the middle of the nucleoid, and at least some of them extend all the way out to the cell envelope. If not separated from the associated protein upon cell lysis, intact chromosomal DNA is trapped by the nucleoid scaffold and precipitates with cell debris, while scaffold-free DNA (like plasmids) stays in solution,—which is used in plasmid isolation protocols based on the precipitation of the chromosomal DNA as a nucleoid. To get insights into the nucleoid structure, we decided to use such a protocol for ‘total plasmid DNA preparation’ (49), to isolate nucleoid-free pieces of the chromosomal DNA (behaving like plasmid DNA) that would separate from the nucleoid as a result of CN + HP-induced CCF.

**Figure 4.**
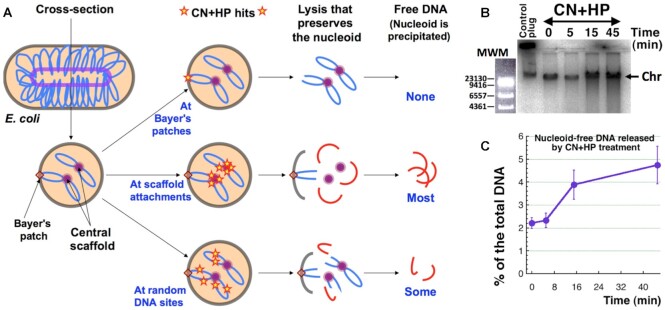
Linear DNA release from the nucleoid by CN + HP treatment. (**A**) A scheme of double-strand break positioning versus linear DNA release from the nucleoid. Brown-walled beige-filled elliptoid, a cell; blue coiled line, DNA duplex; light-purple inner elliptoid that gives two circles in cross-section, the central proteinaceous scaffold. In the middle column, positions of CN + HP-induced double-strand breaks are indicated by small red stars. The nucleoid-free DNA (the red segments in the rightmost column) is isolated and quantified. (**B**) A representative agarose gel image demonstrating the linear DNA release from nucleoid after CN + HP treatment in growing WT cultures. To provide a reference for the total chromosomal DNA, at time zero, the total DNA from 1/10th of the culture volume is collected in an agarose plug, and the total signal from both the well and the lane is determined (the ‘control plug’). In all other lanes, DNA is isolated by the ‘total plasmid DNA’ protocol. Chr, the chromosomal DNA longer than 30 kb, which runs as a single band in 1.1% agarose, independently of its actual length. MWM, molecular weight markers: phage lambda DNA cut with HindIII and stained with ethidium bromide. (**C**) Kinetics of linear DNA release from the nucleoid during CN + HP treatment from several gels like in ‘B’.

We considered three possibilities. CN + HP-induced breaks could target the DNA attachments to the cytoplasmic structures, of which at least two types are known in *E. coli*. All DNA loops must be attached by their bases at the central scaffold (32). In addition, apexes of at least some loops also interact with the cell envelope at specific sites called Bayer's patches (68) (Figure 4A). Interestingly, Bayer's patches are absent in stationary cells (68), and the resistance of stationary WT cells to the CN + HP treatment via Dps-dependent suppression of double-strand breaks (14) could be a reflection of preferential DNA breakage at Bayer's patches in growing cells. The third possibility were random breaks anywhere in protein-free DNA.

Remarkably, the three types of double-strand break positions (random vs. at the central scaffold vs. at the envelope) predict distinct patterns of DNA release from the nucleoid complex (Figure 4A). Double-strand breaks at Bayer's patches should release no DNA from the nucleoid, because all DNA remains attached at the central scaffold (Figure 4A, top row). If, instead, double-strand breaks happen only at the central scaffold, then a substantial amount of DNA is expected to be released (because only two breaks per loop is enough to release the entire loop, and only some of them may be still attached at the envelope) (Figure 4A, middle row). Finally, random breaks should release less DNA from the nucleoid (because attachment sites of both types still function), the extent of ‘random break’ release being dependent on the density of breaks and the density of scaffold attachments (Figure 4A, bottom row).

To see how much short chromosomal DNA pieces would be freed from the nucleoid complex by CCF, we isolated DNA from CN + HP-treated cells using the ‘total plasmid DNA’ protocol (49) at the indicated time-points (Figure 4B) and ran it in a regular agarose gel. The *total DNA* isolated (as before (47)) from 1/10 of the culture volume in agarose plugs at time 0 min was used for normalizing the amount of broken DNA released from the nucleoid. The amount of chromosomal DNA recovered by the total plasmid DNA protocol from untreated control cells is invariable and comprises ∼2% of the total DNA signal (Figure 4B, C). At the same time, after 45 min of treatment with CN + HP, ∼5% of the total genomic DNA is detected as nucleoid-free (Figure 4B, C)—therefore, a mere ∼3% of the chromosomal DNA loses its association with the scaffold as a result of CCF. Taking into account the above reasoning (Figure 4A), as well as the fact that there are ∼50 major DNA loops in a single nucleoid (35,36,39,42), and that 45 min of CN + HP treatment results in a density of at least 100 double-strand breaks per genome equivalent (16), this low yet reproducible level of DNA release is most consistent with DNA breaks at random protein-free positions (Figure 4A, the bottom row). However, the unexpectedly small amount of DNA released from the nucleoid by such a staggering number of double-strand breaks indicates either a much higher density of scaffold-DNA attachments, than is suggested by the pictures of the released nucleoid (32,33,37,40,41), or maybe a different nucleoid structure.

### Testing the nucleoid structure model

**Figure 5.**
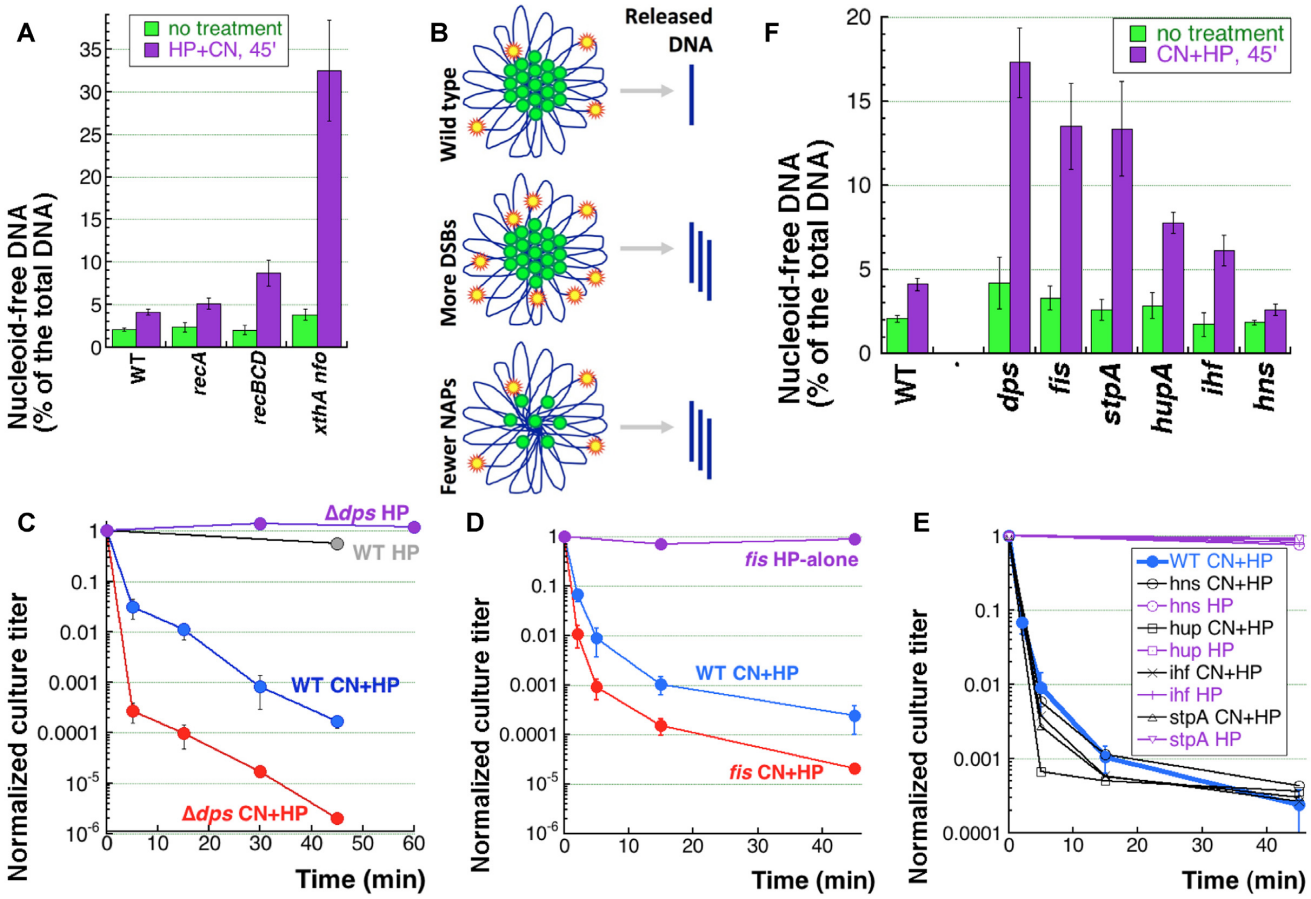
Nucleoid-associated proteins (NAPs) interfere with DNA release from the nucleoids. (**A**) Nucleoid-free DNA in three DNA repair mutants after 45 min of CN + HP treatment. (**B**) Either more DSBs or fewer NAPs should both lead to more nucleoid-free DNA. The nucleoid cross-section features the central scaffold made of NAPs in green, with DNA loops in navy. Double-strand breaks are shown by yellow-red stars. Top, WT situation; middle, more DSBs, e.g. in *xthA nfo* mutant; bottom, fewer NAPs, like in NAP mutants. (**C**) Kinetics of death of WT cells and the *dps* mutant when treated with HP-alone or with CN + HP in growing cultures (14). (**D**) Kinetics of death of WT cells and the *fis* mutant when treated with HP-alone or with CN + HP in growing cultures. (**E**) Kinetics of death of WT cells and the *hns, hupA, ihf* or *stpA* mutants lacking the major nucleoid-organizing proteins when treated with HP-alone or with CN + HP in growing cultures. (**F**) The indicated mutants were treated with CN + HP for 45 min, and the level of nucleoid-free DNA was determined, like in panel ‘A’.

The current model envisions the nucleoid organization as a rosette of multiple DNA loops around the central proteinaceous scaffold (26,27,69). If the DNA release from the nucleoid is due to random breaks within these (mostly naked) DNA loops, then increase in break density should release more DNA from the nucleoid. We tested three DNA repair mutants: 1) *recA* that shows the same level of double-strand breaks in response to CN + HP treatment, 2) *recBCD*, which shows somewhat higher levels of double-strand breaks, 3) the base-excision-repair-deficient *xthA nfo* mutant that suffers ∼10-times more breaks (16). We found that the *recA* mutant releases 2.8% of chromosomal DNA (which is not different from 2% released by WT in this experiment), the *recBCD* mutant releases 6.7%, while the hyper-breaking *xthA nfo* mutant releases almost 30% of the total DNA from its nucleoid (Figure 5A). Thus, increasing the break density by impairing DNA repair does make more DNA nucleoid-free (Figure 5B). Accordingly, the small amount of DNA released in the WT cells (and the *recA* mutants) is because the break density is much lower than the scaffold attachment density. Therefore, by increasing the number of breaks,—for example, by using the base-excision-repair-deficient *xthA nfo* mutant,—the gap between the two densities can be significantly reduced.

Some of these multiple scaffold attachments of the chromosomal DNA should be due to NAPs,—the small and copious proteins isolated with bacterial nucleoid (26,69). Although in comparison with the chromatin organization of the eukaryotic chromosomes the bacterial chromosomal DNA is considered ‘naked’ (27,32,37), from the known number of various NAPs (69) it can be calculated that there is, on average, one NAP molecule per 50 bp of bacterial DNA in growing cells. The six major NAPs in *E. coli*: HU, H-NS, IHF, Fis, StpA and Dps,—are present in various quantities depending on the growth phase and belong to three distinct types depending on their DNA binding mode and function (26,69). The three ‘benders’—HU, Fis and IHF—bind DNA as dimers bending it at significant angles. The two ‘loopers’, H-NS and a related StpA, tend to polymerize along AT-rich DNA and form bridges between different DNA regions, looping out DNA in between. Finally, Dps mostly provides insurance against oxidative damage, as its main function in growing cells is to collect free iron when HP levels rise in the cytoplasm; at the same time, in deep stationary cells Dps becomes the major NAP and packs the chromosome. We have previously reported that the *dps* mutant is more sensitive to CN + HP than WT (14) (Figure 5C), which was expected from its iron-collecting function. Out of the other five NAP mutants, only *fis* showed an increased sensitivity to CN + HP (Figure 5D), while the remaining four individual mutants: *hns, hup, ihf* and *stpA*, all showed WT-like sensitivity (Figure 5E).

We expected the *dps* mutants to release more DNA after CN + HP treatment, because of the higher density of double-strand breaks in this mutant (14), and indeed, the *dps* mutant released almost 14% (Figure 5F). Interestingly, the *fis* mutant, that is also more sensitive to CN + HP, also releases more chromosomal DNA—close to 10% (Figure 5F). In contrast to the *dps* mutant, the effect of the *fis* mutant is likely due to fewer scaffold-DNA attachments (Figure 5B). Even though the same CN + HP sensitivity of the other four individual NAP mutants predicted a WT-like behavior in the DNA release test, three of the mutants, *stpA, hupA* and *ihf*, released 5–10% of the chromosomal DNA (Figure 5F). Thus, even though we have an alternative explanation for the *dps* mutant (more DSBs due to its inability to sequester free iron), the observed increase in the DNA released in the five NAP mutants is probably due to their lower densities of scaffold-DNA attachments (Figure 5B), as most NAPs likely participate in the attachment of the chromosomal DNA to the central scaffold (27).

Interestingly, the *hns* mutant shows an almost 3-fold reduction in the DNA release, which could mean that CN + HP-induced double-strand breaks target DNA : H-NS contacts (there is also a different suggestion below). Overall, we conclude that NAPs do participate in the chromosomal DNA contacts with the scaffold (so their removal increases DNA release). At the same time, CN + HP-induced double-strand breaks do not target chromosomal DNA contacts with various NAPs, with a possible exception of H-NS. And most importantly, significant amount of DNA in the nucleoid stays protein-free,—because increasing the density of the breaks releases significantly more DNA from the nucleoid.

### ATP-dependent DNA release reveals the role of transcription

**Figure 6.**
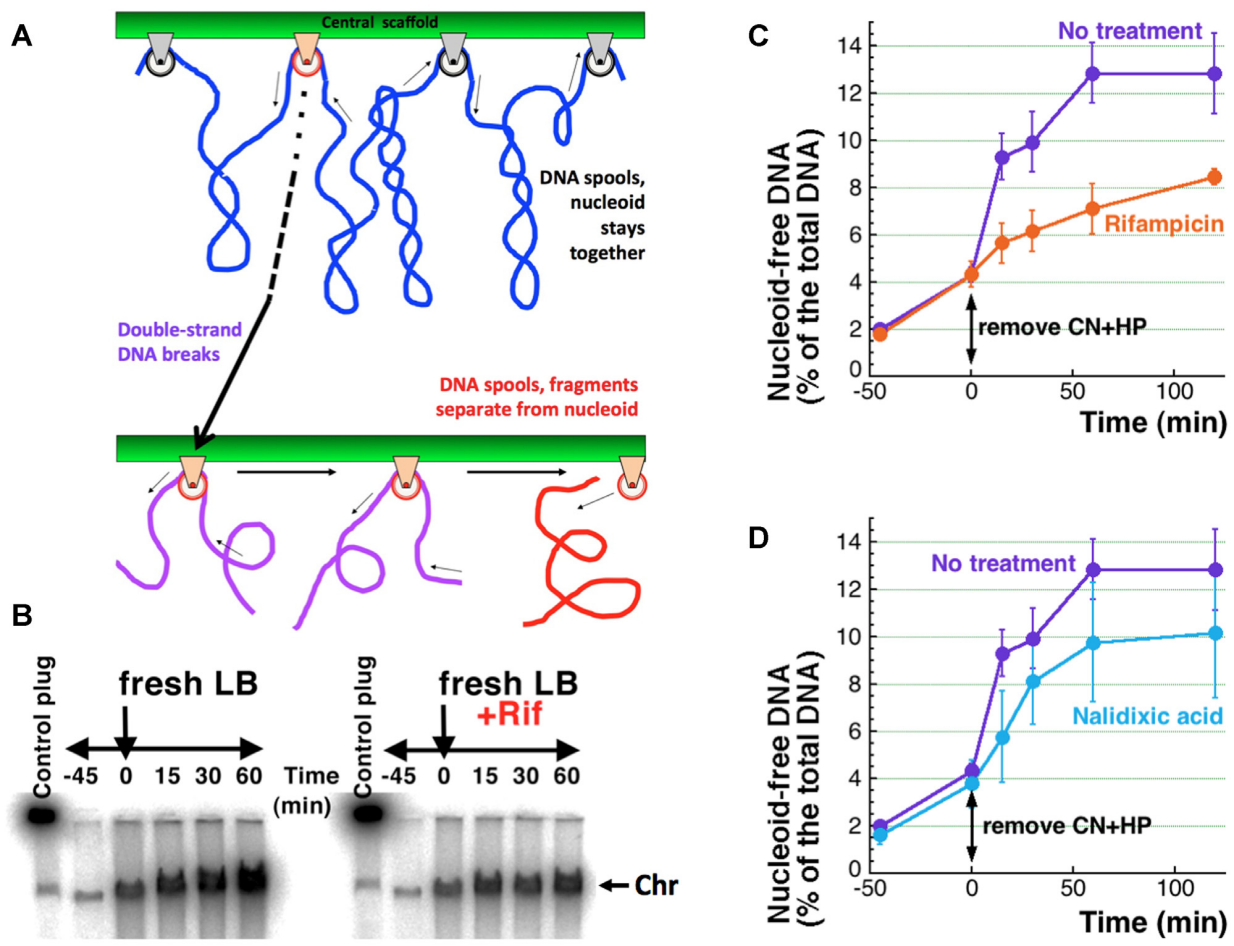
CN + HP removal stimulates further linear DNA release from the nucleoid. (**A**) Top, a scheme of the nucleoid scaffold organization with DNA duplexes (blue) like cables threading through protein pulley blocks, attached at the central scaffold. The direction of threading is shown by thin arrows. One pulley block in the top row is marked red; in the bottom row, the effect of double-strand DNA breaks only at this pulley block is shown. Bottom, upon double-strand DNA breaks (purple cords denote linear DNA fragments), postulated scaffold dynamics releases linear DNA from the pulley block (red cord denotes free DNA). (**B**) A representative agarose gel image showing further release of linear DNA from nucleoid upon removal of CN + HP from WT cells treated for 45 min, and the effect of 100 μg/ml rifampicin added 5 min prior to removal of CN + HP. (**C**) Kinetics of the linear DNA release from several gels like in ‘B’. (**D**) Like in ‘C’, but the effect of 100 μg/ml nalidixic acid added 5 min prior to removal of CN + HP.

Although the structure of the nucleoid as a rosette of similar-sized radial loops (Figure 5B) looks static and suggests designated attachment sites on the chromosomal DNA separating independently-supercoiled domains (loops), several attempts to find such designated domain boundaries, or in fact any short-range periodicity in the nucleoid structure suggesting such boundaries, have failed (70). Instead, four separate 0.5–1.0 Mb-size macrodomains, each bundling several dozens of individual DNA loops, but restricting interaction of macrodomain DNA with any outside DNA, were identified (71–73). At the same time, no internal structure within the macrodomains is detected, perhaps reflecting their highly dynamic behavior in vivo (30). A plausible explanation of how the highly uniform and regularly patterned structure of the nucleoid (32) may still lack stable periodicity, is the transient nature of scaffold attachment to particular DNA stretches (Figure 6A). Accordingly, chromosomal DNA is being continually pulled through these interlocks with the scaffold, sliding like a cable through a pulley box, with no lasting contact of any particular DNA stretch with any particular protein of the central scaffold (Figure 6A, top). Intact chromosomal DNA never disconnects from the scaffold as a result of this sliding, but a broken DNA could, if the pulling continues (Figure 6A, bottom).

In thinking about a possible nature of the pulling force, we came across an alternative explanation for the reduced DNA released in the *hns* mutant. It turns out, *hns* mutants initiate spurious transcription in the middle of genes (74,75)—meaning significantly more transcribing RNA polymerases on the chromosome. Thus, by analogy with eukaryotes, where chromosome scaffold is in contact with highly-transcribed regions (76,77), one of the nucleoid scaffold factors in direct contact with DNA could be transcribing RNA polymerases. If so, they would provide the force and direction to the chromosomal DNA pulling thought the scaffold contacts. But transcription requires energy, and energy production was inhibited in the CN + HP-treated cells. In other words, restoration of ATP-production in the CN + HP-treated cells could release more broken DNA from the nucleoid, due to the resumed transcription.

Transfer of the CN + HP-treated cells to fresh LB restores their ATP production, as demonstrated by the resumed degradation of linear DNA and damaged ribosomal RNA (Figure 2), but will this also ‘unfreeze’ the nucleoid? Transferring WT cells into a fresh medium after CN + HP treatment indeed released an additional ∼9% of the total DNA within one hour, with about half of this amount being released within the first 15 min (Figure 6B, C). Furthermore, blocking transcription initiation with rifampicin reduces the amount of broken DNA released in one hour after ATP restoration to about 1/3 of the uninhibited level (Figure 6B, C), confirming the central role of transcription in this double-strand break-promoted nucleoid disassembly. At the same time, blocking DNA gyrase with nalidixic acid failed to significantly change the kinetics or the final yield of the released DNA (Figure 6D), showing no involvement of DNA supercoiling in this process (which makes sense, as the chromosomal DNA is mostly broken after the CN + HP treatment). We conclude that active transcription provides the force that pulls DNA through the DNA-scaffold contact points, making the contacts dynamic.

### More factors of ATP-dependent DNA release

**Figure 7.**
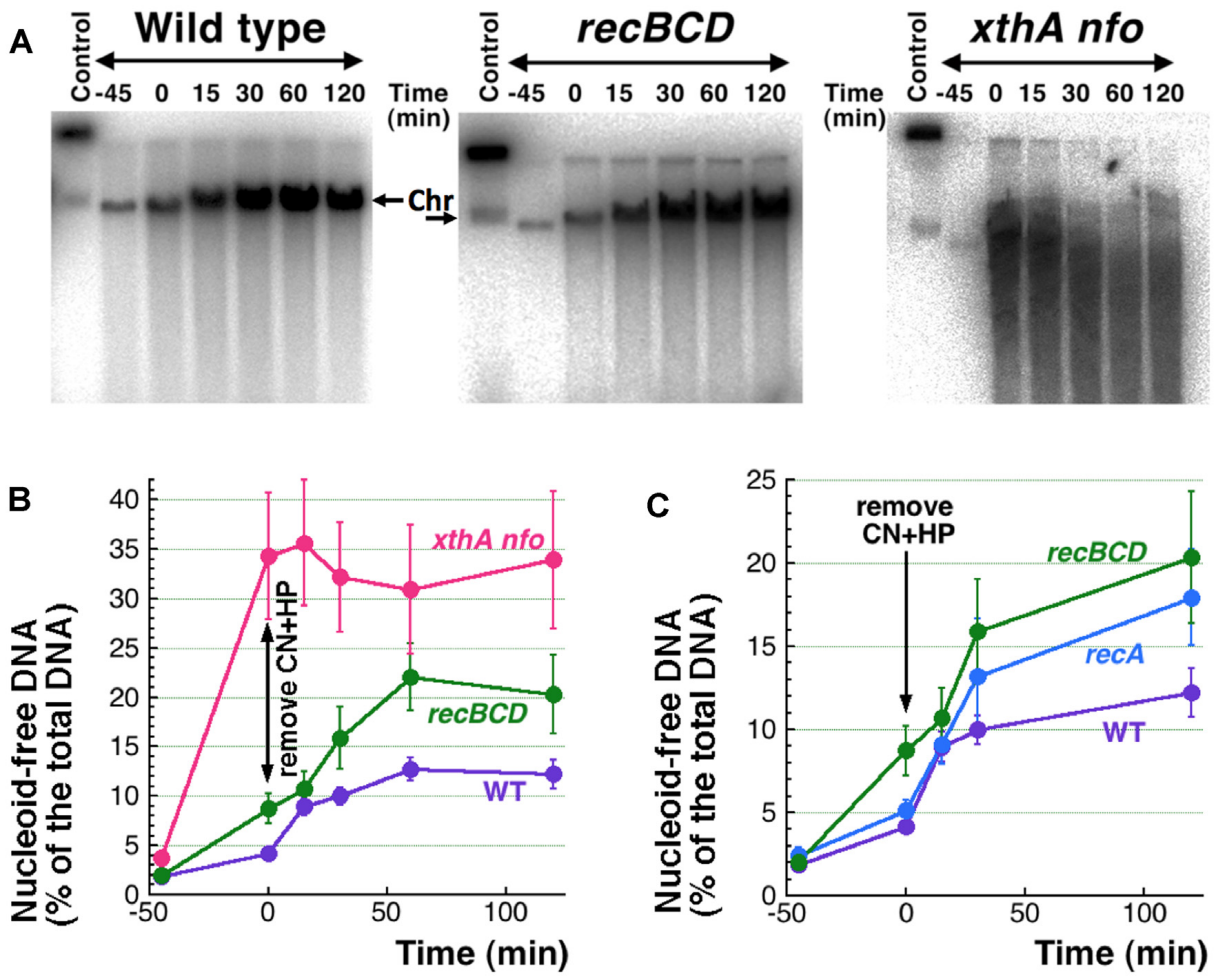
Linear DNA release after CN + HP removal in DNA repair mutants. (**A)** A representative agarose gel image showing further release of linear DNA upon removal of CN + HP, after 45 minute treatment, from cultures of WT, *recBC* or *xthA nfo* mutants. (**B**) Quantification of results from several gels like in ‘A’. (**C**) Quantification of results for the *recA* mutants. The WT and *recBCD* mutant curves are from ‘B’, with the 60 min time point omitted.

Next we measured the ATP-dependent release of broken DNA in mutants lacking recombinational repair of double-strand breaks (even though no repair was detectable in WT cells during the 45 minute CN + HP treatment (16)). Both *recA* and *recBCD* mutants show slightly higher than WT and remarkably similar time courses of ATP-driven DNA release (Figure 7C). In contrast, while the base-excision repair mutant *xthA nfo* releases 10-fold more DNA during the CN + HP treatment itself, this extremely high DNA release does not continue further when ATP becomes available (Figure 7A, B). Thus, the patterns of the original versus ATP-dependent DNA release could be different depending on the DNA repair defect.

**Figure 8.**
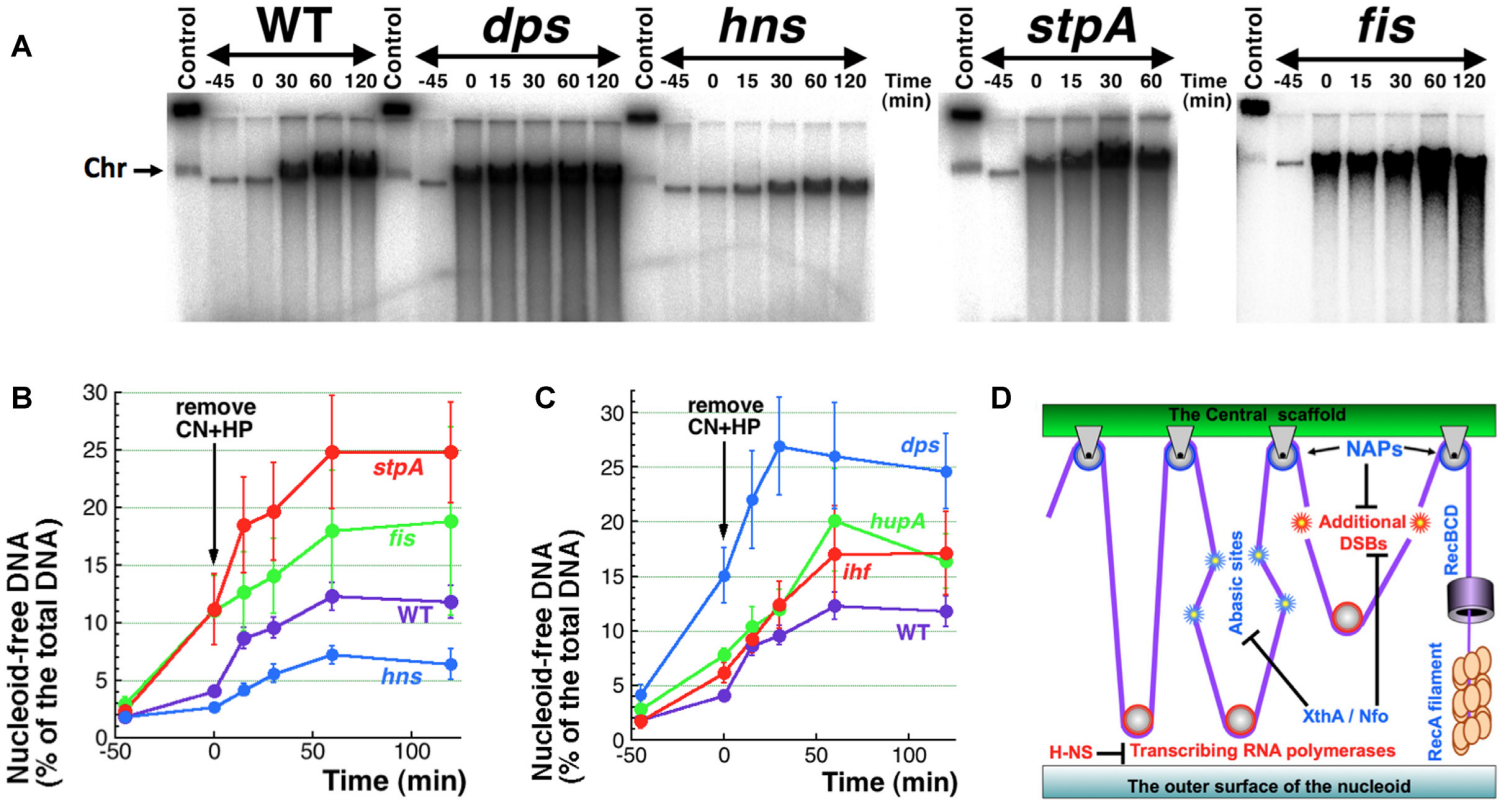
Linear DNA release after the CN + HP removal in NAP mutants. (**A**) A representative agarose gel image showing further release of linear DNA upon removal of CN + HP (after 45 minute treatment) from cultures of WT, *dps, hns, stpA* or *fis* mutants. (**B**) Quantitative kinetics of the results from several gels like in ‘A’. (**C**) Like in ‘B’, but for *dps*, *hupA* and *ihf* mutants. **(D**) A scheme showing processes and proteins/enzymes that either enhance (red) or inhibit (blue) linear DNA release from the nucleoid. DNA (purple line) is duplexed everywhere except in the portion below the RecBCD cylinder, where it is single-stranded.

We also measured the extent and kinetics of ATP-dependent DNA release in mutants lacking individuals NAPs. The only mutant that reduced the WT amounts of released DNA was again *hns* (Figure 8AB), suggesting that the Hns protein either facilitates active DNA sliding through the scaffold pulleys or reduces the number of these pulleys. The other five individual NAP mutants all showed somewhat higher ATP-dependent DNA release compared to WT levels, but the effect was actually modest, considering that they start at different levels after the treatment itself. While the *hupA* and *ihf* mutants start close to WT (Figure 8C), the *dps, stpA* and *fis* mutants start at much higher levels,—leading to the apparently more significant DNA release (Figure 8B, C).

We conclude that multiple factors, including NAPs, double-strand break density and DNA repair proficiency, influence both the immediate (during the CN + HP treatment), as well as the subsequent ATP-dependent DNA release from the nucleoid structure due to CCF. On the one hand, we have identified two factors: the density of double-strand breaks and transcription, and one nucleoid-associated protein, Hns,—which work to increase DNA release. On the other hand, we have also found DNA lesions, proteins and processes that interfere with this ‘nucleoid disassembly via DNA sliding’—namely, abasic sites, nicks, StpA, Fis, HU, IHF NAPs, as well as recombinational repair of double-strand breaks.

## DISCUSSION

Catastrophic chromosome fragmentation (CCF) kills *E. coli* treated with a combination of cyanide (CN) and hydrogen peroxide (HP) at concentrations of the two chemicals that individually are only bacteriostatic (14,16). In this work, we further characterized certain DNA aspects of CCF and its relation to cell killing and to the nucleoid structure. In particular, we show that another known HP-potentiator, nitric oxide (NO) (3,5), as well as a suspected potentiator azide (AZ), in combination treatments, NO + HP and AZ + HP, also induce chromosome fragmentation, with variable intensities and kinetics (Figure 1CD). Importantly, both the killing and chromosome fragmentation by these treatments correlate with each other. Also, for NO + HP, both require intracellular free iron (Figure 1EF)—therefore, are caused by the Fenton reaction. Thus, potentiated HP toxicity via chromosome fragmentation is a general phenomenon.

Focusing on the CN + HP treatment, we next tested the possibility that the observed massive double-strand DNA breakage after this treatment is an artifact of either DNA isolation procedure or pulsed-field gel electrophoresis (PFGE)—and showed that breaks actually happen inside the treated cells, rather than during subsequent manipulations. In aerobically-growing cells, the broken DNA is stabilized by the CN-caused depletion of ATP (61), which blocks linear DNA degradation by RecBCD; by changing the treated cells into a fresh medium to remove CN we were able to jump-start fragmented chromosome degradation in vivo (Figure 2). Mechanistically, we found that the CN + HP-induced double-strand breaks are independent of DNA replication and are rather uniformly distributed over the chromosome (Figure 3).

Using the power of CCF phenomenon to inquire into the nucleoid structure, we then showed that: (i) CN + HP-induced double-strand breaks happen in naked DNA, rather than at the DNA contacts with the nucleoid scaffold, so that broken DNA mostly stays together with the nucleoid complex (Figure 4); (ii) some of the nucleoid-associated proteins (NAPs) prevent additional DNA breakage, while others are part of the scaffold that holds the nucleoid together (Figure 5); (iii) once the cells are changed into a fresh medium to remove the treatment, a significant fraction of the broken chromosomal DNA dissociates from the nucleoid as a result of ATP-dependent processes, mostly transcription (Figures 6–8). On the basis of our findings, we propose the chromosome in *E. coli* to be a tight proteinaceous complex held together by multiple protein-DNA interlocks. Due to the proposed dynamic interlocking nature of the DNA-scaffold interactions, like DNA ‘cables’ sliding over scaffold structures shaped like pulley blocks (Figures 6A, 8D), contacts of any particular DNA sequence with the scaffold are transient. The motors promoting this directional sliding are most likely transcribing RNA polymerases, which may also act as some pulleys (Figure 8D).

### Nucleoid structure and dynamics

Perhaps the most surprising finding of our study was how the supposedly ‘naked’ and ‘loose’ bacterial chromosomal DNA, organized as a rosette of ∼50 megaloops (35,36,39,42), turned out to represent a tight nucleoid structure in the cell. The structure is so solid, that at least 100 (and maybe up to 1,000—Pooja Agashe and A.K., unpublished) double-strand breaks per chromosome release a mere 2–3% of the chromosomal DNA (Figures 4C, 5A). One explanation for this could be that the number of the DNA-scaffold contacts is significantly more than the ∼50 DNA loops detected with the classic approaches,—perhaps attesting to the proposed complex RNA-supported structure of these loops (78,79). According to more recent measurements, the number of independently-supercoiled domains is more than 400 in *E. coli* and *Salmonella*, about 10 kb of DNA per domain (37,38). This increased number of DNA-scaffold contacts is consistent with our observation that increasing the density of DSBs about 10 times increases the release proportionally to ∼30% of the total DNA (Figure 5A). The direct release is also higher in all but one NAP mutant (Figure 5F); although without accurate measurements of the density of breaks it is impossible to rule out more breaks in certain mutants (and there *are* more breaks in the *dps* mutants (14)). Regardless, the increased DNA release in at least some of these mutants is likely due to fewer DNA-scaffold contacts and looser nucleoid.

Returning ATP-production to the treated WT cells releases 9% more of their chromosomal DNA, with two-thirds of the ATP-dependent release during the first hour ascribed to transcription (Figure 6C). Besides identifying a possible motor for DNA sliding over the scaffold interlocks, this result makes transcribing RNA polymerases part of the ‘extended’ nucleoid scaffold. Indeed, transcribing RNA polymerases are present in big quantities in the isolated nucleoids (34,80); if detected in live cells, RNA polymerases are distributed preferentially at the nucleoid periphery (81,82). Again, five out of six individual NAP mutants showed slightly higher ATP-dependent release than WT cells, suggesting that the corresponding proteins act to interfere with the DNA-scaffold sliding in WT cells—in other words, they all participate in the DNA compacting within the nucleoid. The only mutant that showed reduction of ATP-dependent DNA release is *hns* (Figure 8B). As already mentioned, in addition to the obvious possibility of double-strand break targeting to H-NS:DNA contacts, the effect of H-NS absence could be due to a higher density of spurious transcription on the chromosomal DNA in this mutant (74,75)—in the language of our model, it translates into higher densities of pulleys along the DNA cable (Figure 8D).

The other half of the DNA release phenomenon is related to DNA lesions and DNA repair enzymes and complexes. Obviously, double-strand breaks promote DNA release, (Figure 5A); in contrast, accumulation of abasic sites (the substrates of the XthA and Nfo enzymes) apparently interferes with both transcription and DNA sliding through the scaffold attachments, as the *xthA nfo* mutant shows no ATP-dependent DNA release (Figure 7B). The interference is probably due to the kinking that abasic sites cause in the duplex DNA structure (83). The limitation of double-strand break-promoted nucleoid disassembly by activities of double-strand break repair (the stronger ATP-dependent DNA release in the *recA* and *recBCD* mutants (Figure 7C)) likely reflects RecBCD-promoted RecA filament assembly at the broken ends in preparation for their subsequent repair (2,62)—the bulky RecA filament should present a steric block to the end release via sliding off the scaffold contacts (Figure 8D). However, successful double-strand break repair should also reduce DNA release.

## CONCLUSION

Analyzed by atomic force microscopy, gently isolated nucleoids have an unexpectedly smooth globular surface (65), perhaps organized by transcribing RNA polymerases, which within live cells are detected mostly at the nucleoid periphery (81,82), and the associated transcripts. The multiple factors and mechanisms we have identified that are involved in the release of broken DNA from the nucleoid are shown in the overall scheme of Figure 8D. Nucleoid administration is a complicated matter, because, not only the chromosome has to be accommodated in a compartment (i.e. the cell) that is 1000-times shorter than the chromosome length, but also this 1,000-fold compacted chromosome has to support cell growth (i.e. metabolism) via transcription, as well as cell division via DNA replication and segregation. Our work suggests that the nucleoid structure has evolved to keep the compacted chromosome functional, apparently via multiple protein-DNA contacts that are transient for any DNA sequence yet structured in such a way that they can be dissociated only when the DNA experiences a catastrophic number of double-strand breaks. The characterization of these apparently interlocking DNA-protein arrangements involved in the nucleoid administration should be the focus of future studies.

## DATA AVAILABILITY

The authors affirm that all data necessary for confirming the conclusions of the article are present within the article, figures, and tables.

## Notes

Present address: Tulip Mahaseth, 4D Molecular Therapeutics, Emeryville, CA 94608, USA.
